# The *SOD* Gene Family in Tomato: Identification, Phylogenetic Relationships, and Expression Patterns

**DOI:** 10.3389/fpls.2016.01279

**Published:** 2016-08-30

**Authors:** Kun Feng, Jiahong Yu, Yuan Cheng, Meiying Ruan, Rongqing Wang, Qingjing Ye, Guozhi Zhou, Zhimiao Li, Zhuping Yao, Yuejian Yang, Qingsong Zheng, Hongjian Wan

**Affiliations:** ^1^Key Laboratory of Marine Biology, College of Resources and Environmental Science, Nanjing Agricultural UniversityNanjing, China; ^2^State key Laboratory Breeding Base for Zhejiang Sustainable Pest and Disease Control, Institute of Vegetables, Zhejiang Academy of Agricultural SciencesHangzhou, China; ^3^College of Chemistry and Life Science, Zhejiang Normal UniversityJinhua, China

**Keywords:** tomato, superoxide dismutase, *SOD* gene family, promoter, abiotic stress, gene expression

## Abstract

Superoxide dismutases (SODs) are critical antioxidant enzymes that protect organisms from reactive oxygen species (ROS) caused by adverse conditions, and have been widely found in the cytoplasm, chloroplasts, and mitochondria of eukaryotic and prokaryotic cells. Tomato (*Solanum lycopersicum* L.) is an important economic crop and is cultivated worldwide. However, abiotic and biotic stresses severely hinder growth and development of the plant, which affects the production and quality of the crop. To reveal the potential roles of *SOD* genes under various stresses, we performed a systematic analysis of the tomato *SOD* gene family and analyzed the expression patterns of *SlSOD* genes in response to abiotic stresses at the whole-genome level. The characteristics of the *SlSOD* gene family were determined by analyzing gene structure, conserved motifs, chromosomal distribution, phylogenetic relationships, and expression patterns. We determined that there are at least nine *SOD* genes in tomato, including four Cu/ZnSODs, three FeSODs, and one MnSOD, and they are unevenly distributed on 12 chromosomes. Phylogenetic analyses of *SOD* genes from tomato and other plant species were separated into two groups with a high bootstrap value, indicating that these *SOD* genes were present before the monocot-dicot split. Additionally, many *cis*-elements that respond to different stresses were found in the promoters of nine *SlSOD* genes. Gene expression analysis based on RNA-seq data showed that most genes were expressed in all tested tissues, with the exception of *SlSOD6* and *SlSOD8*, which were only expressed in young fruits. Microarray data analysis showed that most members of the *SlSOD* gene family were altered under salt- and drought-stress conditions. This genome-wide analysis of *SlSOD* genes helps to clarify the function of *SlSOD* genes under different stress conditions and provides information to aid in further understanding the evolutionary relationships of *SOD* genes in plants.

## Introduction

It is well known that toxic free radicals caused by environmental stresses such as cold, drought, and water-logging are great challenges for crop production (Mittler and Blumwald, 2010). Among them are reactive oxygen species (ROS), toxic free radicals produced in plant cells in response to stress, which can damage membranes, oxidize proteins, and cause DNA lesions (Sun et al., 2007; Wang and Prabakaran, 2011; Feng et al., 2015). However, in the process of evolution, plants have developed defense mechanisms to alleviate the damage caused by adverse environmental conditions. For example, some well-known ROS-scavenging enzymes can defend plants against environmental stress by controlling the expression of enzyme responsive family genes, including superoxide dismutase (SOD), peroxidase (POD), catalase (CAT), glutathione peroxidase (GPX), and peroxiredoxin (PrxR) (Mittler et al., 2004; Filiz and Tombuloğlu, 2014).

Superoxide dismutases, a group of metalloenzymes, were first found in bovine erythrocytes in Mann and Keilin (1938). Subsequently, they were also described in bacteria, higher plants, and vertebrates (Rabinowitch and Sklan, 1980; Tepperman and Dunsmuir, 1990; Kim et al., 1996; Zelko et al., 2002). McCord and Fridovich (1969), researchers found that SODs can catalyze the dismutation of the superoxide $O_{2}^{–}$ to O_2_ and H_2_O_2_ (Tepperman and Dunsmuir, 1990). In plants, SODs have been detected in roots, leaves, fruits, and seeds (Giannopolitis and Ries, 1977; Tepperman and Dunsmuir, 1990), where they provide basic protection to cells against oxidative stress.

Based on their metal cofactors, protein folds, and subcellular distribution, SODs are mainly categorized as Cu/ZnSODs, FeSODs, and MnSODs (Alscher and Erturk, 2002; Molina-Rueda et al., 2013; Filiz and Tombuloğlu, 2014; Feng et al., 2015). Cu/ZnSODs can be found in prokaryotic and eukaryotic organisms, were first isolated from *Photobacterium leiognathi* in Puget and Michelson (1974; Deshazer et al., 1994), and are present in the cytoplasm, chloroplasts, and/or the extracellular space in plant cells (Pilon et al., 2011). In mammalian cells, the molecular weight of Cu/ZnSODs is about 32 kDa, and they can be found in cytoplasm, nuclear compartments, and lysosomes (Chang et al., 1988; Keller et al., 1991; Crapo et al., 1992; Liou et al., 1993). FeSODs have been found in plant chloroplasts and cytoplasm (Moran et al., 2003; Miller, 2012). MnSODs, widely present in all major kingdoms, have been observed in eukaryotic mitochondria (Lynch and Kuramitsu, 2000) and can protect mitochondria by scavenging ROS (Moller, 2001). MnSODs also play an important role in promoting cellular differentiation (Wispé et al., 1992; Clair et al., 1994). In addition, a new type of *SOD*, NiSOD, has been reported in *Streptomyces* (Youn et al., 1996). However, no evidence for NiSOD has been found in plants (Felisa et al., 2005).

In recent years, some studies have reported that SODs can protect plants against abiotic and biotic stresses, such as heat, cold, drought, salinity, abscisic acid and ethylene (Wang et al., 2004; Pilon et al., 2011; Asensio et al., 2012; Feng et al., 2015). Under various environmental stress conditions, researchers have found that different types of *SOD* genes have different expression patterns. For example, under drought stress, expression patterns of banana genes *MaMSD1A* and *MaCSD1B* were completely opposite to one another (Feng et al., 2015). Moreover, *SODs* with the same metal cofactor did not always play the same role in different species. For example, while we found that expression of MnSODs was not altered under oxidative stress conditions in *Arabidopsis*, researchers found that MnSOD expressions were changed significantly under salt stress in pea and cold and drought stresses in wheat (Gómez et al., 1999; Wu et al., 1999; Baek and Skinner, 2003). These results show that different *SOD* genes have different expression patterns in plants in response to diverse environmental stresses. Additionally, researchers have also found that alternative splicing and miRNAs may participate in the regulation of *SOD* expression (Srivastava et al., 2009; Lu et al., 2011). To date, the *SOD* gene family has been described in many plant species, including *Arabidopsis thaliana*, *Musa acuminata, Sorghum bicolor*, and *Populus trichocarpa* (Kliebenstein and Last, 1998; Molina-Rueda et al., 2013; Filiz and Tombuloğlu, 2014; Feng et al., 2015).

Tomato is not only an important staple and economic crop, but also plays a vital role as an experimental model plant (Mueller et al., 2005). In previous studies, two SOD-coding cDNA sequences isolated from tomato leaves were identified as Cu/ZnSOD genes and were found to be distributed on different chromosomes (Perl-Treves et al., 1990, 1988). Subsequently, the expression levels of two Cu/ZnSOD genes in tomato organs during leaf growth and fruit ripening were reported (Perl-Treves and Galun, 1991). Further, Perl et al. (1993) found that over-expression of Cu/ZnSODs in potato showed that transgenic plants exhibited increased tolerance to oxidative stress. Recently, great efforts have been made to explore the roles of *SOD* genes in improving tomato tolerance to various environmental stresses, such as heat, salt, drought, cold, and bacteria (Mazorra et al., 2002; Li, 2009; Sasidharan et al., 2013; Soydam et al., 2013; Aydin et al., 2014). In addition, several recent studies have indicated that SOD played an important role of hormone and insecticide stresses. For example, Bernal et al. (2009) detected that the expression level of cytosolic Cu/Zn-SOD was increased after 1 day of auxin treatment for tomato. Chahid et al. (2015) studied the activity of SOD in tomato in response to xenobiotics stresses such as alpha-cypermethrin, chlorpyriphos, and pirimicarb. As described above, *SOD* gene families have been widely implicated in responses to abiotic and biotic stresses in tomato. However, these studies have mainly concentrated on the expression of a single form of SOD enzyme and on changes in the enzymatic antioxidant system under various environmental stresses, and little has been reported on the *SOD* gene family in tomato.

To comprehensively understand the putative roles of *SOD* genes in tomato, a systematic analysis of the *SOD* gene family was necessary at the whole-genome level. Recently, the whole-genome sequence of tomato was made available, which provided opportunities for analyzing the expression patterns and regulation mechanisms of the tomato *SOD* (*SlSOD*) gene family in response to environment stresses. Hence, the objectives of this study were (i) to identify the *SOD* gene family in tomato; (ii) to analyze gene structure, duplication and expression patterns in different tomato tissues; (iii) to illustrate chromosomal locations and phylogenetic relationships with *SODs* from other plants; and (iv) to reveal the regulating mechanisms of the *SlSOD* gene family under abiotic (salt and drought) stress by using real-time fluorescence quantitative PCR (qRT-PCR).

## Materials and Methods

### Identification of SOD Genes in Tomato and Other Plant Species

In this study, two methods were used to identify potential SOD genes in tomato. First, the whole tomato genome was downloaded from Sol Genomics Network (SGN^1^) and a local database was constructed using the software Bioedit 7.0 (Pan and Jiang, 2014). Four SOD amino acid sequences from *Solanum lycopersicum* (AF527880-CuSOD), *S. tuberosum* (EU545469-FeSOD), *Arabidopsis thaliana* (AAM62550.1-MnSOD) and *Musa acuminata AAA Group* (AEZ56248.2-FeSOD) were used as a query against the local tomato amino acid database. Second, Hidden Markov Model (HMM) profiles of Cu/ZnSOD (PF00080), Fe-MnSOD (PF00081, PF02777) were downloaded from Pfam^2^. A BlastP search was performed to retrieve candidate tomato SOD genes. For BlastP, *e*-value was set at 1e^-5^. All redundant putative SOD sequences were excluded. The remaining SOD sequences were examined for copper/zinc and iron/manganese SOD domains by the Pfam server^2^. Physicochemical characteristics of SOD amino acid sequences were predicted by the Protparam tool^3^, including molecular weight (MW), and theoretical isoelectric point (*pI*) (Gasteiger et al., 2005).

In addition, to reveal the evolutionary relationships between *SOD* genes in different plant species, potential *SOD* genes from eight plant species were selected for phylogenetic analysis. Among them, *SOD* genes from four plant species (*Vitis vinifera, Solanum tuberosum, Zea mays*, and *Panicum miliaceum*) were identified using the same method above. The *SOD* genes of the remaining plant species (*Poplar, Arabidopsis thaliana, Oryza sativa*, *Sorghum bicolor*) were derived from previously published studies (Kliebenstein and Last, 1998; Dehury et al., 2012; Molina-Rueda et al., 2013; Filiz and Tombuloğlu, 2014).

### Subcellular Localization, Conserved Motifs, and Gene Structure Analysis of SlSOD Proteins

Subcellular localization of SOD proteins from different plant species was obtained from the ProtComp9.0 server^4^ (Feng et al., 2015). Conserved motif analysis of *SlSOD* genes was performed by the Multiple Em for Motif Elicitation (MEME Suite 4.11.1) server^5^ (Bailey et al., 2009). We used the method described by Feng et al. (2015), except that the number of motifs was set to 8. Intron/exon configurations of *SlSOD* genes were determined via the Gene Structure Display Server^6^, for both coding sequences and genomic sequences (Hu et al., 2015).

### Chromosomal Location and Gene Duplication

Information about chromosomal location of *SlSOD* genes was obtained from the SGN database and gene duplications were identified by the Plant Genome Duplication Database (PGDD)^7^ (Singh and Jain, 2015). Tandemly duplicated *SlSOD* genes were identified according to methods reported by previous researchers (Yang and Tuskan, 2008; Tuskan et al., 2006). Chromosomal locations of the *SlSOD* genes were performed with the MapDraw V2.1 tool based on information from the SGN database (Huang et al., 2012). Sequence similarity of SODs was calculated using the program DNAMAN.

### Phylogenetic Tree Construction of SODs

To investigate the phylogenetic relationships of *SlSOD* genes, a total of 108 SOD protein sequences were identified from nine plant species. Among them, 24 SOD protein sequences were excluded owing to the SOD domains being incomplete. Multiple sequence alignments of the remaining 84 SOD amino acid sequences were performed with ClustalW (Higgins et al., 1996) using default parameters. A phylogenetic tree was constructed using the software MEGA5.04 via neighbor-joining method (Tamura et al., 2011). In the phylogenetic tree, the degree of support for a particular grouping pattern was evaluated using bootstrap (1000 replicates) value (Filiz et al., 2014). The tree was viewed with FigTree (v1.3.1).

### Promoter Sequence Analysis

Regions 1,000 bp upstream from the start codons of each *SlSOD* gene were downloaded from SGN. Then, *cis*-elements in promoters of each *SlSOD* gene were predicted using the PlantCARE server (Postel et al., 2002).

### Expression Patterns of *SlSOD* Genes Based on RNA-seq and Microarray Data

For RNA-seq analysis, transcription data of the genome-wide gene expression of the tomato cultivar Helnz were downloaded from the tomato functional genomics database (TFGD)^8^ (Fei et al., 2011). Ten different tissues were selected: root, leaf, bud, flower, 1-cm fruit, 2-cm fruit, 3-cm fruit, mature green fruit (MG), fruit at the fruit breaking stage (B), and fruit 10 days after the fruit breaking stage (B10). RPKM (Reads Per Kilo bases per Million mapped Reads) values of *SlSOD* genes were log2-transformed (Wei et al., 2012). Heat maps of *SlSOD* genes in different tissues were generated using MeV4.9 software (Singh and Jain, 2015).

In addition, microarray data for salt and drought stresses were downloaded from the TOM2 cDNA array and Affymetrix Tomato Genome Array platform from TFGD (Fei et al., 2011). Probe sets corresponding to *SlSOD* genes were identified using the Probe Match Tool in NetAffx Analysis Center^9^ (Altschul et al., 1997). A BlastN search was performed based on sequence alignment between probe sequences and *SlSOD* sequences. Expression patterns of *SlSOD* genes under salt and drought stresses were viewed using MeV4.9 software (Singh and Jain, 2015).

### Plant Materials and Stress Treatment

Seeds of a tomato breeding strain, *Zhe fen 202*, were germinated on water-saturated filter paper. Before germinating, a 10% hypochlorous acid solution was used to sterilize seeds for 5 min. Then, seeds were washed three times with distilled water. Seedlings were grown on Hoagland nutrient solution, in a controlled chamber (25°C/20°C, day/night, 16 h/12 h light/dark cycle). Upon development of the fourth true leaf, seedlings were cultivated in Hoagland nutrient solution with 150 mM sodium chloride (NaCl) and 18% polyethylene glycol (PEG) for treatment (3 and 12 h), respectively. Leaves were collected and frozen in liquid nitrogen immediately and stored at -80°C. Three biological replications were carried out.

### RNA Extraction and qRT-PCR Data Analysis

RNA extraction was performed using the RNAsimple Total RNA Kit (TIANGEN, Beijing, China) according to manufacturer instructions. Before reverse transcription, the quality of RNA samples was checked by agarose gel electrophoresis. All RNAs were reverse transcribed into cDNA using the FastQuant RT Kit with gDNase (TIANGEN, Beijing, China) according to manufacturer instructions.

Specific primers for qRT-PCR analysis were designed using the GenScript server^10^ (Supplementary Table S1) and were synthesized by Sangon Biotech (Shanghai). The GAPDH gene was used as an internal control (Expósito-Rodríguez et al., 2008). An ABI StepOne real time fluorescence quantitative PCR instrument (Applied Biosystems, American) was used for qRT-PCR analysis. The quality and specificity of primers was determined by the melt curve (Feng et al., 2015). Three independent technical replicates were performed for each of the *SlSOD* genes. The PCR program consisted of an initial denaturation at 95°C for 15 min, followed by 40 cycles of 95°C for 10 s, 55°C for 20 s, and 72°C for 30 s. The relative expression levels were calculated using the 2^-ΔΔCt^ method, and were presented by histogram (Livak and Schmittgen, 2001).

## Results

### Genome-Wide Identification of *SOD* Genes Family in Tomato

A total of nine *SOD* genes, classified into two major groups (Cu/ZnSODs and Fe-MnSODs), were identified in tomato. The former group included four members with a copper-zinc domain (*SlSOD1, 2, 3*, and *4*); the latter was composed of five members with an iron/manganese SOD alpha-hairpin domain and an iron/manganese SOD, C-terminal domain (*SlSOD5, 6, 7, 8* and *9*) (**Table 1**). The physicochemical analysis showed that the length of amino acid sequences, MW, and *pI* values varied among these SlSOD proteins. The length ranged from 152 to 311AA, MW ranged from 15.3 to 34.6 kDa, and *pI* ranged from 5.38 to 7.13 (**Table 1**). No significant difference in the acid-base properties of SlSOD proteins was observed, except for *SlSOD9*, which was slightly basic. Using the ProtComp9.0 program, subcellular localizations of SlSOD proteins were determined. Among them, two Cu/ZnSODs (*SlSOD1* and *2*) and one Fe-MnSOD (*SlSOD8*) were predicted to localize in the cytoplasm. One Fe-MnSOD (*SlSOD9*) was localized in mitochondrion. The remaining members were localized in the chloroplast.

**Table 1:** The characteristics of SOD genes from *Solanum lycopersicum*.

Gene name	Sequence ID	Chromosome	ORF Length (bp)	Intron number	Length (aa)	MW (KDA)	pI	Predicted Pfam domain	Subcellular prediction by PC
SlSOD1	Solyc01g067740.2	01	459	6	152	15.3	5.47	CZ	Cytoplasm
SlSOD2	Solyc03g062890.2	03	471	6	156	15.9	6.53	CZ	Cytoplasm
SlSOD3	Solyc11g066390.1	11	654	7	217	22.3	6.01	CZ	Chloroplast
SlSOD4	Solyc08g079830.2	08	936	5	311	32.9	6.45	HMA,CZ	Chloroplast
SlSOD5	Solyc06g048410.2	06	750	8	249	27.9	6.6	IMA,IMC	Chloroplast
SlSOD6	Solyc03g095180.2	03	912	8	303	34.6	5.38	IMA,IMC	Chloroplast
SlSOD7	Solyc02g021140.2	02	759	7	252	29.1	6.65	IMA,IMC	Chloroplast
SlSOD8	Solyc06g048420.1	06	483	4	160	17.9	6.41	IMA,IMC	Cytoplasm
SlSOD9	Solyc06g049080.2	06	687	5	228	25.3	7.13	IMA,IMC	Mitochondrion

*MW, Molecular weight; pI, isoelectric points; CZ, copper/zinc superoxide dismutase (SODC); HMA, heavy-metal-associated domain; IMA, Iron/manganese superoxide dismutases alpha-hairpin domain; IMC, Iron/manganese superoxide dismutases, C-terminal domain; PC, ProtComp9.0 server.*

### Conserved Motif Analysis of SlSOD Proteins

**FIGURE 1:**
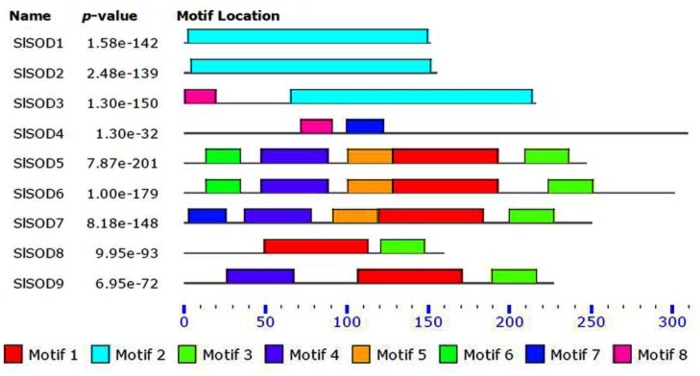
**Conserved motif analysis of SlSOD proteins.** Different color boxes represent different types of motifs.

Four motifs in SlSOD proteins (motif 1 to motif 4) were identified by MEME. Among them, three motifs (motifs 1, 3, and 4) were related to iron/manganese SOD domains, while motif 2 was related to copper/zinc SOD domains (**Table 2**). As shown in **Figure 1**, motif 2 was located in Cu/ZnSODs (*SlSOD1, 2* and *3*), except *SlSOD4*; motif 1 and motif 3 were shared in Fe-MnSODs (*SlSOD5*, *6*, *7*, *8*, and *9*). Motif 4 was widely present in Fe-MnSODs, except for *SlSOD8*.

**Table 2:** Eight different motifs commonly observed in tomato protein sequences by MEME server.

Motif number	Width	Protein sequences	Pfam domain
1	65	ESMKPGGGGEPSGELLQLINRDFGSYDTFVKEFKAAAATQFGSGWAWLAYKPEDKRLAIVKTPNA	IMC (shorten)
		KAVAVLNGNDNVQGTIQFTQDDDGPTTVNGRITGLAPGLHGFHIHALGDTTNGCMSTGPH	CZ (SODC)
2	149	FNPNKKDHGAPMDEVRHAGDLGNIVAGPDGVAEITITDMQIPLTGPHSIIGRAVVVHADPDDLGKGGHE
		LSKTTGNAGGRIACGVIGLQ	
3	28	WEHAYYLDFQNRRPDYISIFMEKLVSWE	IMC (shorten)
4	42	KFDLPPPPYPMDALEPHMSRRTFEFHWGKHHRAYVDNLNKQI	IMA (shorten)
5	28	IILVTYNNGNPLPPFNNAAQAWNHQFFW	
6	22	FLPPQGFNESCRSLQWRTQKKQ	
7	23	CCCQRCVSAVKSDLWRQFGIPNV	
8	20	METHSIFHQTSSDNGFVYPE	

*Among them, Pfam domain of four mitifs (motif5, 6, 7 and 8) were not identify by Pfam server. CZ, copper/zinc superoxide dismutase (SODC); IMA, Iron/manganese superoxide dismutases alpha-hairpin domain; IMC, Iron/manganese superoxide dismutases, C-terminal domain.*

### Chromosomal Distribution and Intron/Exon Configurations of *SlSOD* Genes

**FIGURE 2:**
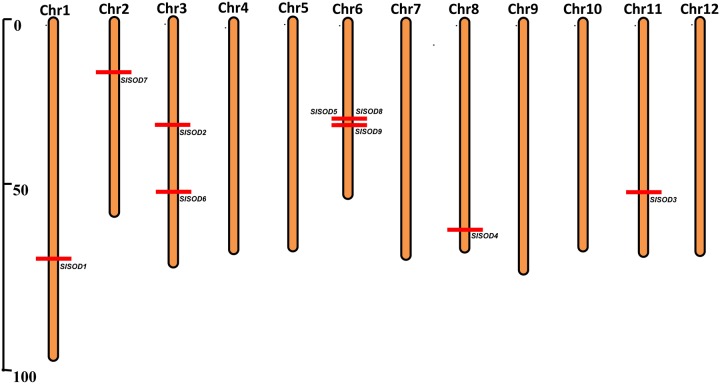
**Chromosomal locations of 9 *SlSOD* genes on 12 chromosomes of tomato.** Red lines represent the position of *SlSOD* genes on chromosomes. The chromosome numbers are indicated at the top of chromosomes. The segment duplication event occurred between SlSOD5 (chromosome 6) and SlSOD6 (chromosome 3) and one cluster including tandemly duplicated genes (SlSOD5 and SlSOD8) on chromosome 6.

The chromosomal distributions of nine *SlSOD* genes were determined. As shown in **Figure 2**, six out of the twelve chromosomes harbored *SlSOD* genes. Chromosome 6 and 3 possessed three and two *SlSOD* genes, respectively, while each of the remaining four chromosomes (chromosome 1, 2, 8, and 11) contained only one *SlSOD* gene. Notably, chromosome 6 had one gene cluster (*SlSOD5* and *8*), which was identified as tandem duplication event. Segmental duplication was identified between *SlSOD5* (chromosome 6) and *SlSOD6* (chromosome 3) by PGDD database. However, despite the sequence similarity between *SlSOD5* and *SlSOD6*, no tandem-duplicated paralogous genes were found in the region surrounding *SlSOD6*.

**FIGURE 3:**
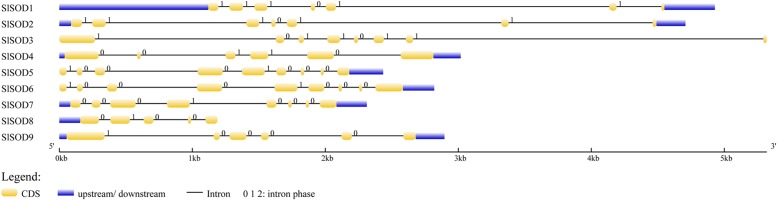
**Intron/exon configurations of *SlSOD* genes.** Exons and introns are shown as yellow boxes and thin lines, respectively. UTRs are shown with blue boxes. 0 = intron phase 0; 1 = intron phase 1; 2 = intron phase 2.

Intron/exon configurations of *SlSOD* genes were constructed using the Gene Structure Display Server (GSDS 2.0^11^) by aligning the cDNA sequences with the corresponding genomic DNA sequences (**Figure 3**). We found that intron numbers among these *SlSOD* genes ranged from 4 to 8. Two *SlSOD* genes (*SlSOD5* and *6*) exhibited the highest intron number (8), whereas *SlSOD8* only had four introns (**Table 1**). In addition, two groups of *SlSOD* genes (*SlSOD1* and *2*, *SlSOD5* and *6*) exhibited similar intron/exon organization patterns, respectively. The rest of the *SlSOD* genes exhibited diverse intron/exon organization patterns.

### Phylogenetic Analysis of *SOD* Genes in Plants

**FIGURE 4:**
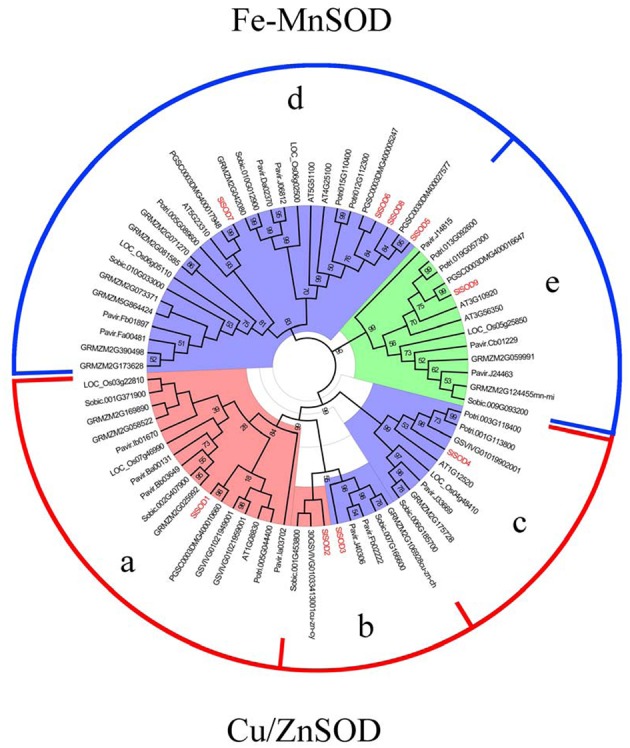
**Phylogenetic tree of 84 SOD proteins from tomato and other plants including *Populus trichocarpa, Arabidopsis thaliana, Vitis vinifera, S. tuberosum, O. sativa, Zea mays, Sorghum bicolor*, and *Panicum miliaceum*.** Two groups (Cu/ZnSODs and Fe-MnSOD) were identified and the tree was divided into five groups (a, b, c, d and e) based on high bootstrap values. SlSOD proteins are marked in red. Three colors (coral, light blue and light green) represent the subcellular locations of SOD proteins: coral represents proteins localized in the cytoplasm, light blue represents proteins localized in chloroplasts, and light green represents proteins localized in mitochondria.

To investigate the phylogenetic relationships of SOD proteins between tomato and other plant species, a total of 108 SOD proteins were identified from *S. lycopersicum*, *Populus trichocarpa, Arabidopsis thaliana, Vitis vinifera, S. tuberosum, O. sativa, Zea mays, Sorghum bicolor*, and *Panicum miliaceum.* Among them, 24 SOD proteins were excluded due to incomplete SOD domains. An unrooted phylogenetic tree was constructed based on the remaining 84 SOD proteins using the program MEGA5.04 (Tamura et al., 2011). As shown in **Figure 4**, the SOD proteins from different plant species were divided into two major groups, Cu/ZnSODs and Fe-MnSODs. The former group was subdivided into three subgroups (a, b, and c), and the latter was separated into two subgroups (d and e), which was strongly supported by high bootstrap values.

In our study, phylogenetic analysis showed that *SlSOD1* grouped with *OsSOD1* (*Loc-Os03g22810*), *OsSOD2* (*Loc-Os07g46990*) and other plants’ cytosolic Cu/ZnSODs clustered in subgroup a. *SlSOD4* grouped with *OsSOD3* and other plants’ chloroplastic Cu/ZnSODs clustered in subgroup c, while two *SlSOD* genes (*SlSOD2* and *SlSOD3*) were clustered with other plants’ cytosolic Cu/ZnSODs and chloroplastic Cu/ZnSODs in subgroup b, respectively. These results indicated that diversity in the Cu/ZnSODs gene family occurred before the splitting of mono- and dicot plants. Interestingly, Cu/ZnSODs genes in subgroup a were separated into mono- and dicot-specific branches, which suggested that they evolved independently after the splitting of mono- and dicot plants. In addition, FeSODs and MnSODs from different plant species were separated by a high bootstrap value (95%). Four genes (*SlSOD5*, *6*, *7*, and *8*) were clustered with other plants’ chloroplastic FeSODs in subgroup d and *SlSOD9* was clustered with other plants’ mitochondrial MnSODs in subgroup e.

### Analysis of *Cis*-Elements in Putative *SlSOD* Gene Promoters

**FIGURE 5:**
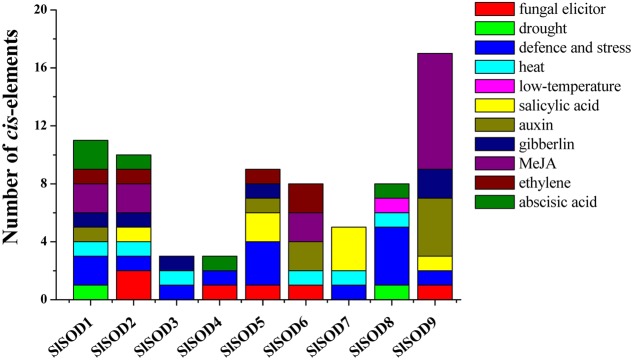
***Cis*-elements in the promoters of putative *SlSOD* genes that are related to stress responses.** Different *cis*-elements with the same or similar functions are present with the same color.

To further understand gene function and regulation patterns, *cis*-elements in *SlSOD* gene promoter sequences were researched. Regions of 1,000 bp upstream from the start codons of each *SlSOD* gene were determined using PlantCARE. The results showed that the *cis*-elements could be divided into three major classes: stress-responsive, hormone-responsive, and light-responsive. Six stress-responsive *cis*-elements were identified, including HSE, MBS, LTR, TC-rich, ARE and Box-W1, which reflected plant responses to heat, drought, low-temperature, defense stresses, anaerobic induction and fungal elicitors, respectively. Ten kinds of hormone-responsive *cis*-elements were identified (e.g., salicylic acid-SA, methyl jasmonate-MeJA, gibberellins-GA, auxin-IAA, and ethylene) (**Figure 5**). A relatively large number of light-responsive *cis*-elements in *SlSOD* promoters was observed (Supplementary Table S2).

### Expression Analysis of *SlSOD* Genes in Different Tissues

**FIGURE 6:**
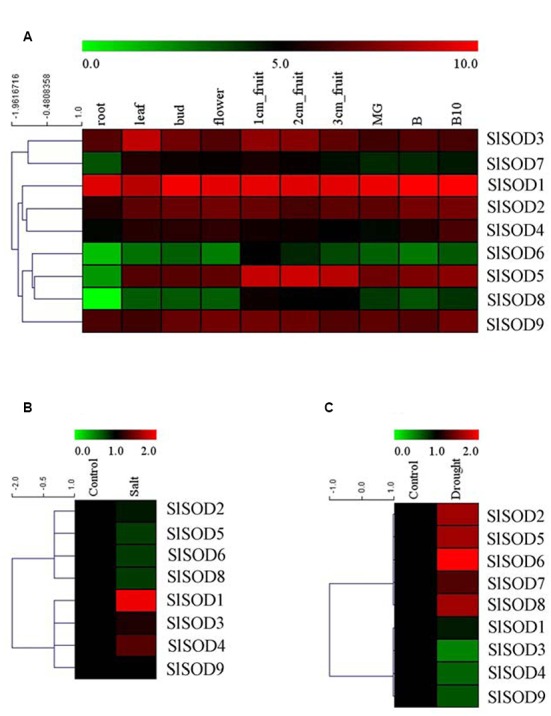
**Expression profiles of *SlSOD* genes in different tomato tissues and under various biotic and abiotic stresses. (A)** The expression patterns of *SlSOD* genes in different tissues. The tested tissues are: root, leaf, bud, flower, 1 cm- fruit, 2 cm- fruit, 3 cm-fruit, mature green fruit (MG), fruit breaking (B), 10 days after fruit breaking (B10). **(B)** Expression profiles of *SlSOD* genes under salt stress. **(C)** Expression profiles of *SlSOD* genes under drought stress.

To explore the expression patterns of *SOD* genes during tomato growth and development, expression profiles were analyzed for 10 different tissues (root, leaf, bud, flower, 1-cm fruit, 2-cm fruit, 3-cm fruit, MG, B, and B10) of the tomato cultivar Helnz using the RNA-seq atlas. As shown in **Figure 6A**, five *SlSOD* genes (*SlSOD1*, *2*, *3*, *4* and *9*) had similar expression patterns in all the tested tissues, while two genes (*SlSOD6* and *8*) displayed distinct tissue-specific expression patterns. Interestingly, *SlSOD1* demonstrated a consistently high expression in all ten tissues, whereas *SlSOD6* and *8* were mainly expressed in young fruit. In addition, *SlSOD7* was expressed strongly in young fruit, weakly in root and moderately in the other tissues.

### Expression Patterns of *SlSOD* Genes in Response to Abiotic Treatments

To gain further insight into the role of *SlSOD* genes under abiotic stress, we analyzed the expression profiles of *SlSOD* genes in response to salt and drought stresses using microarray data. A total of 17 independent tomato microarray probes were identified by means of a BlastN search. As shown in **Figure 6B**, expression of *SlSOD* genes was significantly altered under different abiotic stress treatments. For the salt treatment, eight probes corresponding to *SlSOD* genes were found, with the exception being *SlSOD7*. Expression of four *SlSOD* genes (*SlSOD2*, *5*, *6*, and *8*) were down-regulated, and expression of three *SlSOD* genes (*SlSOD3*, *4*, and *9*) remained constant. Notably, *SlSOD1* was significantly up-regulated. For the drought treatment, microarray probes for each *SlSOD* gene were identified. Four *SlSOD* genes (*SlSOD2*, *5*, *6*, and *8*) were up-regulated and three (*SlSOD3*, *4*, and 9*)* were down-regulated (**Figure 6C**). Notably, *SlSOD6* expression increased twofold. *SlSOD1* and *SlSOD7* were unchanged. We found that the expression levels of most of the *SlSOD* genes changed significantly under salt and drought stresses.

### Verification of *SlSOD* Gene Expression Patterns with qRT-PCR

**FIGURE 7:**
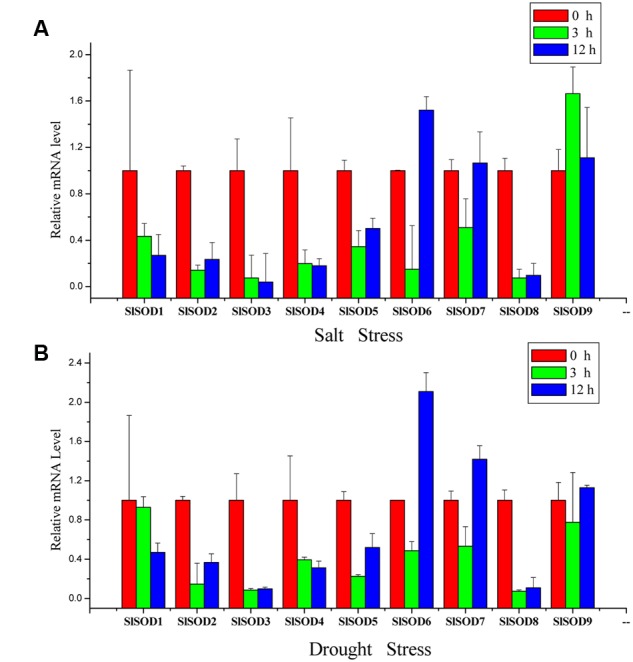
**Gene expression profiles of *SlSOD* genes in response to salt and drought treatments using qRT-PCR. (A)** Expression patterns of *SlSOD* genes under salt stress conditions. **(B)** Expression patterns of *SlSOD* genes under drought stress condition. Error bars represent standard deviations from three independent technical replicates.

To further verify the expression profiles of *SlSOD* genes determined by microarray data analysis, qRT-PCR was used to analyze expression patterns of the nine *SlSOD* genes under salt and drought stresses. As shown in **Figure 7**, most of the *SlSOD* gene results were consistent with the microarray patterns. In response to salt treatment, expression levels of most *SlSOD* genes were down-regulated, in accordance with the microarray profiles. However, expression patterns of three *SlSOD* genes (*SlSOD1*, *6* and *9*) determined by qRT-PCR analysis were different than those determined using the microarray profiles. We found that in response to salt treatment, there was enhanced expression of *SlSOD6* and *SlSOD9* and a decreased transcript level of *SlSOD1*. In response to drought treatment, expression levels of five *SlSOD* genes (*SlSOD1, 3, 4, 6*, and *7*), were consistent with the microarray data. However, in contrast to our microarray results, qRT-PCR analysis showed down-regulation of three *SlSOD* genes (*SlSOD2*, *5*, and *8*) and up-regulation of *SlSOD9*.

## Discussion

Environmental stresses pose considerable challenges for crop production. Gene expression and SOD enzyme activities are influenced by environmental stresses such as high salinity, drought and metal toxicity (Schützendübel and Polle, 2002; Atkinson et al., 2013). However, plants have evolved defense mechanisms to alleviate the damage caused by adverse environmental conditions. Tomato, an important staple and economic crop, is affected by various abiotic stresses (Mueller et al., 2005). SODs are key enzymes in many oxidation processes, and provide basic protection against ROS in plants (Alscher and Erturk, 2002). Therefore, a systematic analysis of the *SlSOD* gene family was performed and gene expression patterns were determined for plants under various abiotic stresses.

In this study, nine *SlSOD* genes (four Cu/ZnSODs, four FeSODs, and one MnSOD) were identified in the tomato genome, including all three major types of plant SOD genes. Chromosome location analysis revealed that *SlSOD5* and *SlSOD8* formed a gene cluster on chromosome 6 and identified as tandem duplicated event. Segmental duplication was identified between *SlSOD5* (chromosome 6) and *SlSOD6* (chromosome 3) by PGDD. Although *SlSOD5* and *SlSOD6* have similar sequence, no tandem duplicated events were occurred in the region surrounding *SlSOD6*. Considering the lower sequence similarity of *SlSOD5* to *SlSOD6* than that of *SlSOD5* to *SlSOD8*, it appears that segmental duplication events predate the tandem duplication in the *SlSOD5* and *SlSOD8* gene cluster (Supplementary Table S3). Therefore, we concluded that segmental duplication and tandem duplication played key roles in the expansion of SOD genes in the tomato genome.

Gene structure analysis revealed that the intron number of *SlSOD* genes ranges from 4 to 8. Previous researchers reported that intron patterns of plant *SOD* genes were highly conserved, and that all cytosolic and chloroplastic *SOD* genes included seven introns except for one member (Fink and Scandalios, 2002; Filiz and Tombuloğlu, 2014). However, in this study, we observed that the intron numbers of six out of eight *SlSOD* genes (excepting *SlSOD9*) were varied, and only two *SlSOD* genes (*SlSOD3*, chloroplastic Cu/ZnSOD and *SlSOD7*, chloroplastic FeSOD) included seven introns. Thus, our data did not support the previous reports of plant *SOD* gene intron patterns. Variation in exon-intron structures was accomplished by three main mechanisms (exon/intron gain/loss, exonization/pseudoexonization and insertion/deletion), each of which contributed to structural divergence (Xu et al., 2012; Filiz and Tombuloğlu, 2014). Moreover, two groups of *SlSOD* genes (*SlSOD1* and *2*, *SlSOD5* and *6*) exhibited similar intron/exon organization patterns, respectively, which suggested high conservation in the evolutionary process.

Phylogenetic analysis showed that FeSODs and MnSOD from different plant species were separated by a high bootstrap value (95%). This result was in agreement with previous report (Fink and Scandalios, 2002). In plants, MnSODs had 70% homology but were different from FeSODs, which suggested that they originated from different ancestral genes (Miller, 2012). These data were well support our results that FeSODs and MnSOD of tomato were diverged with 95% bootstrap value.

To better understand the role of *SlSOD* genes under various environmental stresses, *cis*-elements in the promoter sequences were predicted by PlantCARE server. The results showed that three major classes of *cis*-elements were identified, including stress-responsive, hormone-responsive, and light-responsive. Many identified *cis*-elements in the promotes of *SlSOD* genes were related to heat, drought, low-temperature, defense stresses, anaerobic induction, fungal elicitors, SA, MeJA, GA, IAA and ethylene. As previously stated, *cis*-elements play an important role in plant stress responses; *cis*-elements such as ABRE, DRE, CRT, SARE and SURE respond to abscisic acid (ABA), dehydration, cold, SA, and sulfur, respectively (Sakuma et al., 2002; Maruyama-Nakashita et al., 2005; Shi et al., 2010; Osakabe et al., 2014). Thus, these results will contribute to further understand the various function role of *SlSOD* genes under complex abiotic stress conditions.

To further clarify the potential functions of *SlSOD* genes, expression profiles of all the *SlSOD* genes in different tissues were analyzed. Based on RNA-seq, nine *SlSOD* genes exhibited two disparate expression patterns: constitutive and tissue-specific expression patterns of *SlSOD* genes. During tomato growth and development, two genes (*SlSOD1* and *9*) sustained high expression in all the tested tissues. Two genes (*SlSOD6* and *8*) demonstrated a tissue-specific expression pattern, being mainly expressed in young fruits. *SlSOD7* was expressed strongly in young fruit tissue, weakly in root tissue, and moderately in the rest of the tissues tested.

Under various natural conditions, plant growth and development are frequently affected by high salinity, drought, cold, bacteria, and insecticides (Xia et al., 2012). Previous researchers reported that three types of SODs (Cu/ZnSODs, FeSODs and MnSODs) have been exploited to eliminate ROS caused by abiotic stress (Kliebenstein and Last, 1998; Wu et al., 1999). To further clarify the putative roles of SOD genes in tomato response to abiotic stresses, we examined the expression patterns of nine *SlSOD* genes under salt and drought treatment conditions using microarray and qRT-PCR.

The results showed that the expression patterns of most *SlSOD* genes obtained by qRT-PCR were in conformity with those obtained from the microarray analysis. Expression analysis revealed that most *SlSOD* gene expression levels were changed in response to two abiotic stresses (salt and drought). For salt treatment, *SlSOD1* was the only gene that showed significant up-regulation among the nine *SlSOD* genes, demonstrating that the function of *SlSOD1* relates to salt stress. Compared to the other *SlSOD* genes, a greater variety and quantity of *cis*-elements were found in the promoter for *SlSOD1*, including two TC-rich motifs, an HSEs motif, and an MBS motif, which had been demonstrated to responsible for abiotic stress in banana (Feng et al., 2015). This could explain why *SlSOD1* expression changed significantly under salt treatment. Moreover, although many *cis*-elements related to abiotic stresses were also found in the *SlSOD8* promoter, *SlSOD8* expression was significantly down-regulated under salt treatment. This suggested that some unidentified *cis*-element could play a vital role in regulating the expression of the *SOD* gene family in tomato under abiotic stress. Similar results have also been found in other plant species (Singh and Jain, 2015).

Additionally, we observed different expression patterns of *SlSOD* genes under salt and drought stress conditions (**Figure 6**). For example, the expression of four *SlSOD* genes (*SlSOD2*, *5*, *6*, and *8*) decreased during the salt treatment, while increased expression levels were observed for these four genes in response to drought treatment. This suggested that different *SOD* genes in tomato could play different roles in eliminating ROS caused by different environment stresses.

Taken together, the data we obtained provides more information about the *SOD* gene family in tomato including sequence information, gene duplications, conserved motifs, gene structures, and phylogenetic relationships. Promoter analysis and complex regulation patterns of the *SlSOD* genes under abiotic stress contribute to our understanding of the expression patterns of SOD genes in plants and provide clues for further studies of the roles of *SOD* genes under different stress conditions.

## Author Contributions

Conceived and designed the experiments: HW, QZ, and KF. Performed the experiments: KF, JY, YC, MR, RW, QY, GZ, ZY, and YY. Analyzed the data: KF, JY, and ZL. Wrote the paper: KF and HW. All authors have read and approved the manuscript.

## Conflict of Interest Statement

The authors declare that the research was conducted in the absence of any commercial or financial relationships that could be construed as a potential conflict of interest.

## Footnotes

1 https://solgenomics.net/

2 http://pfam.xfam.org/

3 http://web.expasy.org/

4 http://linux1.softberry.com/

5 http://meme-suite.org/tools/meme

6 http://gsds.cbi.pku.edu.cn/

7 http://chibba.agtec.uga.edu/duplication/

8 http://ted.bti.cornell.edu/

9 http://www.affymetrix.com

10 https://www.genscript.com/ssl-bin/app/primer

11 http://gsds.cbi.pku.edu.cn/index.php
