# Supplementary material for: The SOD Gene Family in Tomato: Identification, Phylogenetic Relationships, and Expression Patterns
Source: Front Plant Sci. 2016 Aug 30;7:1279. doi: 10.3389/fpls.2016.01279 (PMC5003820; doi:10.3389/fpls.2016.01279)
Supplement: Supplementary file 1 [file Table_1.DOC]

Supplementary Table S1 Pairwise similarities among the nine SlSOD genes

|  | SlSOD 1 | SlSOD 2 | SlSOD 3 | SlSOD 4 | SlSOD 5 | SlSOD 6 | SlSOD 7 | SlSOD 8 | SlSOD 9 |
| --- | --- | --- | --- | --- | --- | --- | --- | --- | --- |
| SlSOD 1 | 100 |  |  |  |  |  |  |  |  |
| SlSOD 2 | 63.8 | 100 |  |  |  |  |  |  |  |
| SlSOD 3 | 64.5 | 63.8 | 100 |  |  |  |  |  |  |
| SlSOD 4 | 9.4 | 10.9 | 12.3 | 100 |  |  |  |  |  |
| SlSOD 5 | 15.2 | 13 | 10.9 | 15.2 | 100 |  |  |  |  |
| SlSOD 6 | 16.7 | 13 | 10.9 | 13.0 | 85.5 | 100 |  |  |  |
| SlSOD 7 | 10.1 | 10.9 | 9.4 | 10.1 | 51.4 | 52.2 | 100 |  |  |
| SlSOD 8 | 14.5 | 12.3 | 10.1 | 13.8 | 86.2 | 75.4 | 46.4 | 100 |  |
| SlSOD 9 | 12.3 | 10.9 | 8.7 | 15.9 | 31.2 | 33.3 | 29.7 | 29 | 100 |
