# Supplementary material for: The SOD Gene Family in Tomato: Identification, Phylogenetic Relationships, and Expression Patterns
Source: Front Plant Sci. 2016 Aug 30;7:1279. doi: 10.3389/fpls.2016.01279 (PMC5003820; doi:10.3389/fpls.2016.01279)
Supplement: Supplementary file 2 [file Table_2.DOC]

Supplementary Table S2 The number of *cis*-elements related to various environment stresses in promoter of nine SlSOD genes.

| Gene | Fungal elicitor | Drought | Defense and stress | Heat | Low-  temp | SA | IAA | GA | MeJA | ethylene | ABA | Light |
| --- | --- | --- | --- | --- | --- | --- | --- | --- | --- | --- | --- | --- |
| SlSOD1 | 0 | 1 | 2 | 1 | 0 | 0 | 1 | 1 | 2 | 1 | 2 | 12 |
| SlSOD2 | 2 | 0 | 1 | 1 | 0 | 1 | 0 | 1 | 2 | 1 | 1 | 14 |
| SlSOD3 | 0 | 0 | 1 | 1 | 0 | 0 | 0 | 1 | 0 | 0 | 0 | 6 |
| SlSOD4 | 1 | 0 | 1 | 0 | 0 | 0 | 0 | 0 | 0 | 0 | 1 | 16 |
| SlSOD5 | 1 | 0 | 3 | 0 | 0 | 2 | 1 | 1 | 0 | 1 | 0 | 7 |
| SlSOD6 | 1 | 0 | 0 | 1 | 0 | 0 | 2 | 0 | 2 | 2 | 0 | 6 |
| SlSOD7 | 0 | 0 | 1 | 1 | 0 | 3 | 0 | 0 | 0 | 0 | 0 | 11 |
| SlSOD8 | 0 | 1 | 4 | 1 | 1 | 0 | 0 | 0 | 0 | 0 | 1 | 6 |
| SlSOD9 | 1 | 0 | 1 | 0 | 0 | 1 | 4 | 2 | 8 | 0 | 0 | 14 |

Low-temp: low-tempreture, SA: salicylic acid, IAA: auxin,MeJA: methyl jasmonate, GA: gibberellins, ABA: abscisic acid
