# Supplementary material for: The SOD Gene Family in Tomato: Identification, Phylogenetic Relationships, and Expression Patterns
Source: Front Plant Sci. 2016 Aug 30;7:1279. doi: 10.3389/fpls.2016.01279 (PMC5003820; doi:10.3389/fpls.2016.01279)
Supplement: Supplementary file 3 [file Table_3.DOC]

Supplementary Table S3 Specific primer for qRT-PCR of each SlSOD gene.

| Gene | Forward primer | Reverse primer |
| --- | --- | --- |
| SlSOD1 | AACCTGGACTTCATGGCTTC | CCAGCAGGATTGTAATGTGG |
| SlSOD2 | AATCTCCGGGAACGATAGTG | AAGGCATGGATATGGAAAGC |
| SlSOD3 | CTCCTGGAGATGAAATCCGT | AAGTGCTCGTCCAACAACTG |
| SlSOD4 | CCTCCGATAATGGAGTCGTT | GGTTTGCAATTTGCTCTTGA |
| SlSOD5 | AGCATTCAACAATGCTGCTC | CTCCGTTGGGCTTCATAGAT |
| SlSOD6 | GGCTTGGAACCATCAATTCT | TCAAAGGAGCCGAAGTCTCT |
| SlSOD7 | TGCCTAAATTGGGATTCCTC | CTCTGCTCAAGACAAGCCAA |
| SlSOD8 | AGATGAAGCCTAACGGAGGA | GTTTGTATGCAAGCCAGGAC |
| SlSOD9 | GCAGAAGGTGCTGCTTTACA | AGGCGCTTAAGCTCTTTGTC |
